# High-Risk Cytogenetic Multiple Myeloma Remains a Therapeutic Challenge: A 15-Year Real-World Analysis

**DOI:** 10.3390/curroncol33050249

**Published:** 2026-04-27

**Authors:** Carmel Awadallah, Anas Zayad, Shebli Atrash, Anita Mazloom, Omar Alkharabsheh, Prerna Mewawalla, Mansi R. Shah, Forat Lutfi, Zahra Mahmoudjafari, Muhammad Umair Mushtaq, Jeries Kort, Alma Habib, Al-Ola Abdallah

**Affiliations:** 1 Division of Internal Medicine, St John Episcopal Hospital, Far Rockaway, NY 11691, USA; awadallah@qaffaf.com; 2 Department of Internal Medicine, Hamad Medical Corporation, Doha 3050, Qatar; 3 Levine Cancer Center, Atrium Health, Wake Forest University School of Medicine, Charlotte, NC 28204, USA; 4 Division of Hematology and Oncology, Mitchell Cancer Institute, University of South Alabama, Mobile, AL 36604, USA; 5 Division of Hematology and Oncology, University of Cincinnati, Cincinnati, OH 45219, USA; 6 Division of Hematology and Bone Marrow Transplant, Alleghany Medical Center, Pittsburg, PA 15212, USA; 7 Division of Blood Disorders, Rutgers Cancer Institute, New Brunswick, NJ 08903, USA; 8 Division of Hematologic Malignancies & Cellular Therapeutics, University of Kansas Medical Center, Westwood, KS 66205, USA

**Keywords:** multiple myeloma, high-risk cytogenetics, treatment patterns, real-world evidence, induction therapy, proteasome inhibitor, immunomodulatory drug, autologous stem cell transplantation

## Abstract

**Background**: High-risk cytogenetic multiple myeloma (HRMM) confers inferior outcomes despite significant therapeutic advances. Real-world data characterizing contemporary treatment patterns across lines of therapy in this population are limited. **Methods**: We conducted a multicenter retrospective study of 205 patients with HRMM diagnosed between January 2009 and January 2024. High-risk cytogenetics were defined according to the International Myeloma Working Group (IMWG) and Revised International Staging System (R-ISS) criteria as the presence of del(17p), t(4;14), t(14;16), t(14;20), and/or gain/amplification of 1q21. Treatment regimens were categorized by the number of agents (doublet, triplet, quadruplet), mechanism of action (proteasome inhibitor [PI]-based, immunomodulatory drug [IMiD]-based, and anti-CD38 monoclonal antibody-based), and specific regimen composition. Treatment patterns were analyzed across three treatment eras and stratified by age (<70 vs. ≥70 years). **Results**: The cohort included 205 patients (median age, 62 years [interquartile range [IQR], 54–68 years]; 78.5% aged <70 years). Double-hit and triple-hit cytogenetics were present in 60.0% and 7.3% of patients, respectively. For first-line induction, triplet therapy predominated (81.0%, n = 166), followed by quadruplet (8.8%, n = 18), doublet (5.9%, n = 12), and multi-agent chemotherapy (2.9%, n = 6). By mechanism of action, PI + IMiD combinations were the most common (63.4%, n = 130), followed by PI-based (19.0%, n = 39), anti-CD38-based (10.2%, n = 21), and IMiD-based (2.9%, n = 6) regimens. Era-stratified analysis revealed a significant increase in quadruplet utilization from 3.2% in Era 1 (2009–2015) to 20.3% in Era 3 (2020–2024), with anti-CD38-containing frontline regimens administered to 26.6% of patients in the contemporary era (*p* < 0.001). Second-line induction was required in 50 patients (24.4%), with anti-CD38-based triplets comprising the most common approach (30.0%). Autologous stem cell transplantation (ASCT) was performed in 75.1% of patients overall, with significantly higher utilization in those aged <70 years (83.9% vs. 43.2%). Maintenance therapy was administered to 71.7% of patients (n = 147), with IMiD-based regimens predominating (53.1%), followed by PI + IMiD combinations (27.9%) and anti-CD38-containing regimens (10.2%). Despite intensive therapy, 69.4% of patients on maintenance experienced disease relapse, with higher rates observed in younger patients (74.8% vs. 41.7%). **Conclusions**: In this real-world HRMM cohort, the PI + IMiD triplet regimen remained the predominant induction approach, with a significant shift toward quadruplet and anti-CD38-containing regimens in the contemporary era. However, substantial relapse rates during maintenance underscore the continued unmet need in HRMM and support the early integration of novel therapeutic strategies, including quadruplet induction and BCMA-directed agents, in this population.

## Simple Summary

Multiple myeloma is a blood cancer that remains difficult to treat when patients have specific high-risk chromosomal abnormalities. Despite significant advances in therapy over the past two decades, patients with these high-risk features continue to have worse outcomes compared to standard-risk patients. In this study, we examined real-world treatment patterns in 205 patients with high-risk multiple myeloma treated across multiple US centers over a 15-year period (2009–2024). We found that treatment has evolved substantially, with more patients now receiving four-drug combinations and regimens that include daratumumab, an antibody that targets the CD38 protein on myeloma cells. However, despite these advances, the majority of patients relapsed after maintenance therapy, highlighting that high-risk multiple myeloma remains an unmet medical need. These findings support the earlier use of novel treatment strategies, including quadruplet induction and therapies targeting the BCMA protein, to improve outcomes in this challenging patient population.

## 1. Introduction

Multiple myeloma (MM) is a clonal plasma cell neoplasm characterized by heterogeneous clinical behavior and outcomes [1,2]. Cytogenetic abnormalities represent one of the most powerful prognostic factors in MM and form the foundation of contemporary risk stratification systems [3,4,5]. High-risk cytogenetic abnormalities, including deletion 17p (del (17p)), translocations t(4;14) and t(14;16), and gain or amplification of chromosome 1q21 (gain (1q)), are associated with inferior progression-free and overall survival despite advances in therapy [6,7].

The therapeutic landscape for MM has evolved substantially over the past two decades with the introduction of proteasome inhibitors (PIs), immunomodulatory drugs (IMiDs), CD38-directed monoclonal antibodies, and, more recently, T-cell redirecting therapies [8,9,10,11]. Current guidelines recommend triplet or quadruplet induction regimens incorporating PIs and IMiDs, with the addition of anti-CD38 antibodies increasingly employed in high-risk patients [12,13,14]. However, optimal treatment sequencing and the real-world adoption of guideline-concordant therapy in high-risk populations remain incompletely characterized.

Real-world evidence is essential for understanding treatment patterns, outcomes, and practice variation outside the controlled setting of clinical trials, particularly in high-risk subgroups that are often underrepresented or excluded from pivotal studies [15,16]. Furthermore, real-world data provide critical insights into treatment sequencing, tolerance, and effectiveness in heterogeneous patient populations with comorbidities and performance status limitations commonly encountered in routine practice. Characterizing contemporary practice patterns may identify gaps between evidence-based recommendations and actual clinical implementation, inform quality improvement initiatives, and establish benchmarks for future research.

This study aimed to comprehensively characterize real-world treatment patterns across multiple lines of therapy in patients with high-risk multiple myeloma (HRMM), including first-line induction (including second induction when required), consolidation with autologous stem cell transplantation, and post-transplant maintenance therapy. Secondary objectives included assessing overall response rates to induction therapy, evaluating post-transplant outcomes, and comparing treatment patterns and outcomes between younger (<70 years) and older (≥70 years) patient populations.

## 2. Materials and Methods

### 2.1. Study Design, Population, and Cytogenetic Definitions

This multicenter retrospective cohort study included adults (≥18 years) with high-risk multiple myeloma (HRMM) diagnosed according to the International Myeloma Working Group (IMWG) criteria and evidence of measurable disease. Patients were identified through electronic health records at participating centers between January 2009 and January 2024.

Eligible patients were identified through electronic health records and institutional tumor registries at each participating center. Consecutive adult patients (≥18 years) with newly diagnosed multiple myeloma meeting IMWG criteria and harboring at least one high-risk cytogenetic abnormality on FISH performed on purified plasma cells at diagnosis were included, provided they received at least one line of anti-myeloma therapy at the participating institution.

Patients were excluded if FISH results were unavailable or performed on unpurified bone marrow samples, if the diagnosis could not be confirmed from available records, or if there was insufficient clinical documentation to characterize treatment exposure.

FISH panels were performed at each participating institution according to local laboratory protocols, and panel composition varied across sites and time periods. Cytogenetic data were recorded based on positive findings documented in clinical reports. Negative results were not systematically confirmed, and the absence of a specific abnormality may reflect incomplete panel testing rather than a true negative result. This is particularly relevant for t(14;20) and del(1p), which were documented in fewer than 2% of patients and may therefore be underestimated in this cohort.

High-risk cytogenetics were defined by the presence of one or more abnormalities detected by interphase fluorescence in situ hybridization (FISH) on purified plasma cells, including deletion 17p [del(17p)], t(4;14), t(14;16), t(14;20), gain or amplification of 1q21 [gain/amp(1q)], and deletion 1p [del(1p)], consistent with the IMWG cytogenetic risk-stratification criteria [17,18]. Although the prognostic significance of 1q gain/amplification and del(1p) continues to evolve, these abnormalities were included based on contemporary IMWG-informed frameworks and prior literature. Positivity thresholds were based on institutional FISH assay validation: gain or amplification of 1q21 was defined as present when detected in >4.6% of analyzed plasma cells, t(4;14), t(14;16), and t(14;20) were considered positive when present in >3.0% of plasma cells, and del(17p) was defined as TP53 loss in >20% of plasma cells.

Del(1p) was included in the cytogenetic risk definition based on contemporary IMWG-informed frameworks; however, it was documented in only 4 patients (1.9%) in this cohort, likely reflecting inconsistent ascertainment across participating sites.

Double-hit multiple myeloma (DHMM) is characterized by the presence of two high-risk abnormalities, whereas triple-hit multiple myeloma (THMM) is defined by the presence of three or more high-risk abnormalities [19,20].

### 2.2. Treatment Classification

Treatment regimens were classified using three complementary frameworks to characterize therapeutic patterns across lines of therapy.

First, the regimens were categorized according to the number of active anti-myeloma agents. Doublet therapy consisted of one active agent plus dexamethasone (Vd, Rd, and Kd). Triplet therapy included two active agents plus dexamethasone (e.g., RVd, KRd, and VCd/CyBorD), whereas quadruplet therapy comprised three active agents plus dexamethasone, typically incorporating an anti-CD38 antibody (e.g., Dara-RVd and Dara-KRd).

Second, regimens were grouped according to their pharmacologic backbone. Proteasome inhibitor (PI)–based regimens contained bortezomib, carfilzomib, or ixazomib without an immunomodulatory drug (IMiD). IMiD-based regimens included lenalidomide- or pomalidomide-containing combinations without a PI. PI + IMiD regimens incorporated both classes (e.g., RVd, KRd, KPd, IRd). Anti-CD38 monoclonal antibody–based regimens were defined by the inclusion of daratumumab or isatuximab (e.g., DRd, DVd, DKd, DPd, Dara-RVd, Isa-Kd), regardless of the presence of accompanying PI or IMiD components. Chemotherapy-based regimens included intensive cytotoxic combinations, such as D-PACE, VD-PACE, and KD-PACE. Regimens incorporating novel immune therapies were categorized as “Other,” including belantamab mafodotin, BCMA-directed CAR T-cell therapies (ide-cel, cilta-cel), and BCMA- or GPRC5D-targeted bispecific antibodies (e.g., teclistamab, elranatamab, talquetamab).

For patients who received a brief stabilization or bridging regimen prior to a definitive induction regimen for example, one to two cycles of CyBorD in the setting of acute renal failure prior to transition to VRd the regimen administered with primary disease-control intent was classified as first-line induction. Short bridging courses of ≤2 cycles administered solely for acute stabilization were not considered a separate line of therapy. Second-line induction was defined as a change in regimen prior to ASCT or definitive therapy due to inadequate response, disease progression, or treatment intolerance, as documented in the medical record. This intent-based classification was applied uniformly to all pre-ASCT regimen modifications, as the therapeutic goal in these patients remained to achieve deeper cytoreduction and proceed to transplant consolidation, rather than representing a distinct post-progression treatment phase.

Finally, individual regimens were recorded as administered to enable a granular analysis of treatment patterns. Each regimen was assigned to its principal mechanistic class to ensure consistent categorization across patients and treatment lines.

### 2.3. Outcomes and Assessments

The primary outcome was the characterization of treatment patterns across lines of therapy, including first-line induction, second-line induction (when applicable), autologous stem cell transplantation (ASCT), and post-transplant maintenance. For each line, we recorded the specific regimens, frequency of use, and distribution according to the treatment classification framework described in Section 2.3. Treatment patterns were compared between patients aged <70 and ≥70 years. Temporal trends were evaluated across three treatment eras, Era 1 (2009–2015; PI/IMiD era), Era 2 (2016–2019; early anti-CD38 adoption), and Era 3 (2020–2024; contemporary quadruplet era), to assess changes in regimen selection, anti-CD38 utilization, and quadruplet adoption.

The secondary outcomes included: (1) ASCT utilization, conditioning regimens, and tandem transplantation rates by age group; (2) maintenance therapy patterns, including mechanism-based classification and temporal trends; and (3) relapse rates during maintenance therapy, compared between age groups.

Relapse was defined according to IMWG criteria as recurrence or progression of disease evidenced by reappearance of M-protein, new bone lesions or plasmacytomas, increased bone marrow plasma cells, hypercalcemia, or other myeloma-defining events attributable to plasma cell proliferation [21].

Patients who died without a documented relapse were classified as relapse-free for the purpose of this analysis. Relapse status was unavailable in two patients who were excluded from the relapse proportion calculation.

### 2.4. Statistical Analysis

Descriptive statistics summarized patient characteristics, treatment patterns, and outcomes. Categorical variables are presented as frequencies and percentages, while continuous variables are reported as medians with interquartile ranges (IQR) because of non-normal distributions.

Comparisons between age groups (<70 vs. ≥70 years) were performed using the chi-square test or Fisher’s exact test for categorical variables and the Mann–Whitney U test for continuous variables. Statistical significance was defined as a two-sided *p* < 0.05. Given the exploratory real-world nature of the analysis, no adjustments for multiple comparisons were applied.

To evaluate temporal changes in treatment patterns, exploratory analyses were stratified by the era of diagnosis: Era 1 (2009–2015; PI/IMiD era), Era 2 (2016–2019; early anti-CD38 adoption), and Era 3 (2020–2024; contemporary era with quadruplet induction, frontline anti-CD38 antibodies, and BCMA-directed therapies). Treatment distributions across eras were compared using the chi-square test or Fisher’s exact test.

Missing data were handled using an available-case analysis, with denominators adjusted for evaluable patients. All analyses were performed using IBM SPSS Statistics for Windows, version 28.0 (IBM Corp., Armonk, NY, USA).

### 2.5. Ethical Approval

This study was conducted in accordance with the Declaration of Helsinki and approved by the Institutional Review Board (IRB) of each participating center. The coordinating center IRB approval was obtained under protocol number 00160483, approved on 8 October 2024. The requirement for informed consent was waived due to the retrospective nature of the study and minimal risk to participants. All data were de-identified prior to analysis to protect patient confidentiality, and data handling procedures complied with the Health Insurance Portability and Accountability Act (HIPAA) regulations.

## 3. Results

### 3.1. Baseline Patient Characteristics

We included 205 patients with HRMM in the analysis. The median age at diagnosis was 62 years (interquartile range [IQR] 54–68; range, 33–95 years). Most patients (n = 161, 78.5%) were aged <70 years (median age 60 years, IQR 52–64), while 44 patients (21.5%) were aged ≥70 years (median age 75 years, IQR 72–79).

Demographics and Performance Status: The cohort was predominantly White (n = 131, 63.9%) and African American (n = 56, 27.3%), with a higher proportion of White patients in the ≥70-year subgroup (77.3% vs. 60.2%). Males comprised 51.7% (n = 106) of the population, with a similar distribution across age groups. Most patients had preserved functional status, with an ECOG performance status of 0–1 documented in 80.5% (n = 165) overall; however, an ECOG of 2–3 was more frequent in older patients (29.5% vs. 16.8%, *p* = 0.016).

Disease Characteristics: The Revised International Staging System (R-ISS) distribution showed Stage II disease in 61.0% (n = 125), Stage III in 36.1% (n = 74), and Stage I in 0% (n = 0); 2.9% (n = 6) had unknown staging. The predominant myeloma subtypes were IgG kappa (33.2%, n = 68), IgG lambda (22.9%, n = 47), and IgA kappa (17.1%, n = 35). Light chain-only disease was present in 16.6% (n = 34) of the patients.

Cytogenetic Profile: High-risk cytogenetic abnormalities included gain(1q) in 59.0% (n = 121), del(17p) in 55.1% (n = 113), t(4;14) in 31.2% (n = 64), t(14;16) in 15.6% (n = 32), and t(14;20) in 1.5% (n = 3). Del(17p) was more prevalent among older patients (65.9% vs. 52.2%, *p* = 0.032), whereas t(4;14) was more common in patients aged <70 years (34.8% vs. 18.2%, *p* = 0.043). The standard-risk translocation t(11;14) was present in 7.8% (n = 16) of patients, with a higher frequency in the ≥70 years group (15.9% vs. 5.6%). Double-hit cytogenetics (two concurrent high-risk abnormalities) were present in 60.0% (n = 123) of patients, and triple-hit disease (three or more abnormalities) was identified in 7.3% (n = 15). Extramedullary disease at diagnosis was observed in 16.1% (n = 33) of patients.

The complete baseline characteristics are summarized in Table 1.

### 3.2. First-Line Induction Therapy

All 205 patients received first-line induction therapy (two patients had unknown or unavailable regimen data). Treatment regimens were analyzed according to three complementary classification frameworks: (1) number of active agents, (2) mechanism of action/therapeutic backbone, and (3) specific regimen composition.

#### 3.2.1. Classification by the Number of Agents

Triplet regimens (two active agents plus corticosteroid) were the predominant induction approach used in 166 patients (81.0%), with VRd being the most common regimen (56.1%), followed by CyBorD (15.1%) and KRd (3.9%). The less frequent triplets included DRd (2.0%), VTd (1.5%), and DVd or KCd (1.0% each).

Quadruplet regimens were administered to 18 patients (8.8%), predominantly DVRd (6.8%), whereas a small number of patients received trial-based combinations (elotuzumab/VRd or VRd plus belantamab). Doublet regimens were used in 12 patients (5.9%), most commonly Vd (2.9%), followed by Rd (2.0%), and Td (1.0%). Chemotherapy-based regimens were uncommon (2.9%), and one patient received the BCMA bispecific antibody linvoseltamab.

Across treatment eras, triplet regimens remained the dominant backbone, accounting for 75.8% in Era 1 (2009–2015), 93.8% in Era 2 (2016–2019), and 74.7% in Era 3 (2020–2024) (*p* = 0.015). VRd remained the most common regimen in all eras, although its use declined in Era 3 as quadruplet and anti-CD38-containing regimens emerged. CyBorD use increased over time, whereas KRd was primarily used during Era 2.

Quadruplet utilization increased significantly, rising from 3.2% in Era 1 to 20.3% in Era 3 (*p* < 0.001), largely driven by DVRd adoption following the GRIFFIN and PERSEUS trials. Conversely, doublet use declined substantially from 14.5% in Era 1 to <3% in later eras, reflecting a shift toward more intensive induction strategies (Table 2).

#### 3.2.2. Classification by Mechanism of Action

PI + IMiD combinations (including both triplet and quadruplet formulations) predominated across all eras (Era 1:71.0%, 44/62; Era 2:76.6%, 49/64; Era 3:46.8%, 37/79), with VRd comprising the overwhelming majority (115/130, 88.5% of the PI + IMiD regimens). PI-based regimens without an IMiD backbone were used in 39 patients (19.0%), predominantly CyBorD (31/39, 79.5%). Anti-CD38-containing frontline regimens were absent in Eras 1 and 2 but were utilized in 26.6% (21/79) of patients in Era 3 (*p* < 0.0001), with DVRd being the most common anti-CD38–based regimen (14/21, 66.7%), followed by DRd (4/21, 19.0%), DVd (2/21, 9.5%), and DVCd (1/21, 4.8%). IMiD-based regimens without a PI were rare (6/205, 2.9%), predominantly confined to Era 1 (5/62), with a single case in Era 3. Table 2.

### 3.3. Second Induction Therapy

Fifty patients (24.4%) required a second induction regimen prior to transplant or definitive therapy. The most common reasons were disease progression or inadequate response (40%), followed by treatment intolerance (28%), including lenalidomide-related rash, bortezomib-induced neuropathy, and gastrointestinal toxicity. The remaining 32% switched for other reasons (e.g., renal function changes, bridging strategies, clinical trial enrollment, or undocumented causes).

PI-based regimens were most frequently used (40%), followed by PI + IMiD combinations (28%), most commonly VRd (12%) and KRd (10%). IMiD-based regimens without a PI accounted for 16%, predominantly DPd (10%). Chemotherapy-based regimens were administered in 12%, whereas a small number received sequential multi-regimen approaches.

Younger patients (<70 years) received a broader range of therapies, including PI + IMiD combinations (34.1%) and chemotherapy (14.6%). In contrast, older patients (≥70 years) predominantly received PI-based regimens (77.8%), and none received chemotherapy or quadruplet combinations as second inductions (Table 3).

### 3.4. Maintenance Therapy

Among 147 patients (71.7%) receiving maintenance therapy (76.4% of patients aged <70 years vs. 54.5% of those aged ≥70 years; *p* = 0.004), IMiD-based monotherapy was the most common approach (53.1%), driven predominantly by lenalidomide monotherapy, which accounted for 50.3% of all maintenance-treated patients. Lenalidomide remained the dominant maintenance strategy across eras, although its use declined over time (Era 1:74.0% → Era 2:48.8% → Era 3:29.6%).

PI + IMiD combinations represented the second most common strategy (27.9%), with bortezomib plus lenalidomide being the most frequent (18.4%). The use of these combinations has increased over time (Era 1:14.0% → Era 2:27.9% → Era 3:40.7%), reflecting a shift toward intensified maintenance in the contemporary era.

Anti-CD38-based maintenance was used in 10.2% of cases, primarily daratumumab-based combinations, emerging in Era 2 (7.0%) and expanding in Era 3 (22.2%). Two patients received belantamab mafodotin plus lenalidomide in clinical trials.

PI monotherapy was administered in 7.5% of patients (bortezomib, ixazomib, or Kd) and was used exclusively in patients <70 years, with a marked decline in Era 3.

Among older patients (≥70 years), the most common maintenance strategies were lenalidomide monotherapy (50.0%), PI + IMiD combinations (25.0%), and anti-CD38-based regimens (16.7%), with no use of PI monotherapy (Table 4).

Of the 147 patients receiving maintenance therapy, 133 (90.5%) had previously undergone ASCT and 14 (9.5%) had not. Maintenance regimen selection was broadly similar between groups; however, the small size of the non-ASCT subgroup precluded meaningful formal comparison.

### 3.5. Autologous Stem Cell Transplantation

Of the 205 patients, 154 (75.1%) underwent autologous stem cell transplantation (ASCT), with one patient (0.5%) receiving allogeneic SCT. Transplant utilization differed markedly by age: 83.9% of patients aged <70 years (135/161) underwent ASCT compared with 43.2% of those aged ≥70 years (19/44) (*p* < 0.001).

#### Conditioning Regimen and Tandem ASCT

High-dose melphalan (200 mg/m^2^) was the most common conditioning regimen (60.5% overall), used more frequently in patients <70 years (71.4%) than in those ≥70 years (20.5%). Reduced-intensity melphalan (140 mg/m^2^) was administered in 12.2% of patients, with higher use in older patients (22.7% vs. 9.3%). Rare regimens included melphalan 100 mg/m^2^ and busulfan–melphalan (each 0.5%). Tandem ASCT was performed in 14 patients (6.8%), exclusively among those <70 years. Post-transplant treatment modalities are summarized in Table 5.

### 3.6. Relapse During Maintenance

**Figure 1:**
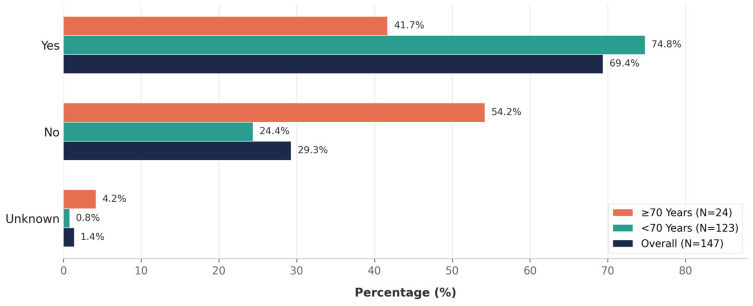
Relapse During Maintenance Therapy by Age Group (<70 vs. ≥70 Years). Among 147 patients on maintenance therapy, 69.4% experienced relapse during follow-up. Relapse rates were markedly higher in patients <70 years (74.8%) compared to those ≥70 years (41.7%), while a greater proportion of older patients remained relapse-free (54.2% vs. 24.4%).

Among 147 patients receiving maintenance therapy, at a median follow-up of 47.5 months from maintenance initiation, relapse occurred in 102 (69.4%), while 43 (29.3%) remained relapse-free; relapse status was unavailable in two patients (1.4%). Relapse was more frequent among patients younger than 70 years (74.8%; median follow-up 58.9 months) compared with those aged 70 years or older (41.7%; median follow-up 27.1 months), whereas a higher proportion of older patients remained relapse-free (54.2% vs. 24.4%) (*p* = 0.003) (Figure 1).

These differences should be interpreted cautiously and may reflect longer follow-up among younger patients, competing mortality risks in older patients, variations in disease biology, or differences in maintenance therapy intensity and adherence.

Despite intensive therapy, including ASCT in ~75% of patients and maintenance therapy in most cases, the high relapse rate (69.4%) highlights the persistent unmet need in HRMM, supporting the earlier integration of novel strategies, such as BCMA-directed therapies, MRD-adapted treatment approaches, and innovative maintenance combinations (Figure 1).

### 3.7. Efficacy Outcomes

Following induction therapy, the overall response rate (ORR) was 83.9% (n = 172). By depth of response, stringent complete response (sCR) was achieved in 4.9% of patients, complete response (CR) in 17.1%, very good partial response (VGPR) in 34.1%, and partial response (PR) in 27.8%, resulting in a combined VGPR or better rate of 56.1%. Stable disease was observed in 1.5% of patients, and 8.3% had primary refractory disease.

Era-stratified post-induction ORR was 91.9% in Era 1 (2009–2015), 82.8% in Era 2 (2016–2019), and 82.3% in Era 3 (2020–2024), with corresponding VGPR-or-better rates of 59.7%, 56.2%, and 53.2%, respectively.

Among the 154 patients who underwent ASCT, the post-transplant ORR was 88.3% (n = 136). Responses deepened following ASCT, with sCR, CR, and VGPR rates of 16.9%, 33.8%, and 33.1%, respectively. The combined CR/sCR rate was 50.6%, and the VGPR or better rate was 83.8%. Progressive disease following ASCT was documented in 7.1% of patients.

Post-ASCT response stratified by age demonstrated that all 19 patients aged ≥70 years who underwent ASCT achieved at least a partial response (100%), with 94.7% achieving ≥VGPR and 63.2% achieving CR/sCR. These outcomes were comparable to or numerically higher than those observed in patients aged <70 years (≥PR 86.7%, ≥VGPR 82.2%, CR/sCR 48.9%).

Post-maintenance response assessment demonstrated that 22.4% of patients experienced disease progression within 12 months of maintenance initiation, as detailed in Table 6.

At a median follow-up of 46.9 months (IQR 24.1–83.1) from diagnosis, the median progression-free survival (PFS) from diagnosis to first relapse or death was 22.0 months overall and was shorter in patients aged ≥70 years (12.2 months) compared with younger patients (25.6 months). Median overall survival (OS) was 46.9 months overall, with age-stratified medians of 49.5 months in patients <70 years and 38.9 months in those aged ≥70 years.

These data demonstrate progressive deepening of response with treatment intensification. The CR/sCR rate increased from 22.0% following first-line induction to 50.6% post-ASCT, consistent with the established consolidative effect of transplant. The high ORR following second-line induction (90.0%) reflects effective salvage prior to transplant. In contrast, the increasing rates of progressive disease at 6 months (15.0%) and 12 months (19.0%) of maintenance highlight the aggressive biology of HRMM and the ongoing unmet need in this population (Table 6).

Response assessment at 12 months of maintenance was unavailable in 29.3% of patients (43/147), reflecting the retrospective design and variability in documentation across sites. This has been acknowledged as a limitation.

## 4. Discussion

This multicenter retrospective study provides 15-year real-world evidence on treatment patterns and outcomes in high-risk cytogenetic multiple myeloma (HRMM) across six US institutions. PI + IMiD triplet combinations remained the predominant frontline induction strategy throughout the study period, achieving a post-induction ORR of 83.9% and a VGPR-or-better rate of 56.1%. ASCT was performed in 75.1% of patients overall, with post-transplant deepening of response to a VGPR-or-better rate of 83.8% and a CR/sCR rate of 50.6% [22]. Despite this intensive approach, 69.4% of maintenance-treated patients relapsed at a median follow-up of 47.5 months, with a median PFS of 22.0 months

A clinically meaningful shift in treatment practice was observed over the three eras. Anti-CD38-containing frontline regimens were absent before 2020 but were used in 26.6% of patients in Era 3 (*p* < 0.001), driven by the adoption of daratumumab-based quadruplets following the GRIFFIN and PERSEUS trials [10,23]. Quadruplet utilization rose from 3.2% in Era 1 to 19.0% in Era 3, consistent with evolving guidelines recommending intensified induction for transplant-eligible patients [12,13,24]. Era-stratified response rates were comparable across periods (ORR 91.9%, 82.8%, and 82.3% for Eras 1, 2, and 3, respectively), though the substantially shorter follow-up in Era 3 (median 25.1 months vs. 97.4 months in Era 1) precludes meaningful OS and PFS comparisons across eras. Longer follow-up of contemporary patients will be necessary to determine whether the shift toward quadruplet and anti-CD38-based regimens translates into improved survival outcomes.

Age-related differences were clinically significant. ASCT utilization was markedly lower in patients aged 70 years or older (43.2% vs. 83.9%, *p* < 0.001), yet older patients who underwent transplantation achieved excellent responses, with 100% attaining at least a PR and a CR/sCR rate of 63.2%, supporting the principle that chronological age alone should not preclude transplant eligibility in fit older adults [25,26]. Maintenance therapy patterns reflected guideline-based practice, with lenalidomide predominating across all eras, though its use declined over time in favor of PI + IMiD and anti-CD38-based combinations [27]. The 22.4% progression rate within 12 months of maintenance and the high double-hit (60.0%) and triple-hit (7.3%) cytogenetic burden highlight the biological aggressiveness of this cohort and the limitations of current maintenance strategies in HRMM.

### 4.1. Comparison with Contemporary Real-World Studies

Our findings are consistent with recent real-world analyses. Rahman et al. reported comparable triplet utilization (~65%) but higher quadruplet adoption (12%), likely reflecting a more recent treatment period [28,29]. The post-induction ORR of 83.9% in our cohort is consistent with pivotal trials such as SWOG S0777 [30], while the post-ASCT CR/sCR rate of 50.6% aligns with rates reported in the DETERMINATION trial [31]. The GMMG-CONCEPT trial reported a 100% ORR with Isa-KRd specifically in HRMM [32], suggesting that further intensification with isatuximab-based quadruplets may yield deeper responses in this population. The persistent relapse rate during maintenance in our cohort, in contrast with the greater than 60% three-year PFS achieved with lenalidomide maintenance in standard-risk disease [25], underscores the need for risk-adapted maintenance strategies in HRMM.

### 4.2. Clinical Implications and Future Directions

Several clinically relevant questions arise from these findings. First, the optimal frontline regimen for specific cytogenetic subgroups remains undefined. Patients with del(17p) may derive particular benefit from daratumumab-based quadruplets based on CASSIOPEIA subgroup analyses [9], while t(4;14) disease may respond preferentially to proteasome inhibitor-intensive strategies. Future studies should evaluate outcomes stratified by individual and combined cytogenetic abnormalities. Second, the low use of BCMA-directed therapies in our cohort (6.8–8.1%), primarily in the relapsed setting [33,34,35], contrasts with emerging evidence supporting earlier integration. The CARTITUDE-4 trial demonstrated significantly improved PFS with ciltacabtagene autoleucel (HR 0.29) and confirmed the first OS benefit for a CAR-T therapy (HR 0.55) [36,37,38,39], supporting a paradigm shift toward earlier use of BCMA-directed agents in HRMM [40]. Third, MRD-adapted treatment strategies and intensified maintenance incorporating PI + IMiD or anti-CD38 combinations warrant prospective evaluation in this high-risk population.

### 4.3. Study Limitations

This study has several important limitations. The retrospective design introduces selection bias, and treatment decisions were physician-directed without standardized protocols, limiting causal inference. The 15-year study period spans major therapeutic shifts, and earlier treatment patterns do not reflect contemporary practice. The multicenter design, while enhancing generalizability, introduces heterogeneity in cytogenetic testing platforms, FISH positivity thresholds, and response assessment practices. Era-stratified survival comparisons are substantially confounded by differential follow-up (median 97.4, 58.2, and 25.1 months across eras), and formal Kaplan–Meier analyses were not performed. Risk stratification was based on conventional FISH, predating the 2025 IMS-IMWG Consensus Genomic Staging framework [41], which may reclassify a proportion of our cohort as standard- or intermediate-risk. MRD data, detailed toxicity profiles, and dose modification records were not systematically available [42]. Finally, the tertiary referral center composition may overestimate ASCT utilization and novel agent exposure relative to community practice.

## 5. Conclusions

In this real-world multicenter cohort of patients with high-risk cytogenetic multiple myeloma, the PI + IMiD triplet regimen was the predominant first-line induction approach, achieving high overall response rates. Autologous stem cell transplantation was performed in three-quarters of the patients, with excellent post-transplant responses. Despite intensive therapy, disease progression during maintenance remained substantial, highlighting the continued unmet need in this high-risk population. Future studies should evaluate the impact of quadruplet induction and novel agents on outcomes in HRMM.

## Figures and Tables

**Table 1:** Baseline characteristics of patients with high-risk multiple myeloma by age group.

Characteristic	Overall (N = 205)	<70 Years (N = 161)	≥70 Years (N = 44)
**Age, Median (IQR; Range)**	62 (54–68; 33–95)	60 (52–64; 33–69)	75 (72–79; 70–95)
**Race**			
White	131 (63.9%)	97 (60.2%)	34 (77.3%)
African American	56 (27.3%)	48 (29.8%)	8 (18.2%)
Asian	7 (3.4%)	6 (3.7%)	1 (2.3%)
Hispanic	4 (2.0%)	4 (2.5%)	0 (0.0%)
Native American	1 (0.5%)	1 (0.6%)	0 (0.0%)
Other	6 (2.9%)	5 (3.1%)	1 (2.3%)
**Sex (Male)**	106 (51.7%)	81 (50.3%)	25 (56.8%)
**ECOG Performance Status**			
0	27 (13.2%)	24 (14.9%)	3 (6.8%)
1	138 (67.3%)	110 (68.3%)	28 (63.6%)
2	32 (15.6%)	23 (14.3%)	9 (20.5%)
3	8 (3.9%)	4 (2.5%)	4 (9.1%)
**R-ISS Stage**			
Stage I	0 (0.0%)	0 (0.0%)	0 (0.0%)
Stage II	125 (61.0%)	97 (60.2%)	28 (63.6%)
Stage III	74 (36.1%)	58 (36.0%)	16 (36.4%)
N/A	6 (2.9%)	6 (3.7%)	0 (0.0%)
**Type of Myeloma**			
Free Kappa	23 (11.2%)	19 (11.8%)	4 (9.1%)
Free Lambda	11 (5.4%)	6 (3.7%)	5 (11.4%)
IgA Kappa	35 (17.1%)	28 (17.4%)	7 (15.9%)
IgA Lambda	19 (9.3%)	14 (8.7%)	5 (11.4%)
IgG Kappa	68 (33.2%)	55 (34.2%)	13 (29.5%)
IgG Lambda	47 (22.9%)	39 (24.2%)	8 (18.2%)
Non-secretory	2 (1.0%)	0 (0.0%)	2 (4.5%)
**Cytogenetics**			
del(17p)	113 (55.1%)	84 (52.2%)	29 (65.9%)
t(4;14)	64 (31.2%)	56 (34.8%)	8 (18.2%)
t(14;16)	32 (15.6%)	25 (15.5%)	7 (15.9%)
t(14;20)	3 (1.5%)	3 (1.9%)	0 (0.0%)
1q gain	121 (59.0%)	97 (60.2%)	24 (54.5%)
t(11;14)	16 (7.8%)	9 (5.6%)	7 (15.9%)
**Double-hit present**	123 (60.0%)	98 (60.9%)	25 (56.8%)
**Triple-hit present**	15 (7.3%)	13 (8.1%)	2 (4.5%)
**EMD**	33 (16.1%)	27 (16.8%)	6 (13.6%)

Abbreviations: ECOG, Eastern Cooperative Oncology Group; R-ISS, Revised International Staging System; N/A, not applicable; EMD, extramedullary disease; IQR, interquartile range. High-risk cytogenetics defined as del(17p), t(4;14), t(14;16), t(14;20), and/or 1q gain per R-ISS and IMWG criteria. t(11;14) classified as standard-risk.

**Table 2:** First-Line Induction Therapy: Classification by Number of Agents and Specific Regimens, Stratified by Treatment Era and Age Group (N = 205).

Category/Regimen	Era 1 (2009–2015) (N = 62)	Era 2 (2016–2019) (N = 64)	Era 3 (2020–2024) (N = 79)	Overall (N = 205)	<70 Years (N = 161)	≥70 Years (N = 44)
**Quadruplet**	**2 (3.2%)**	**0**	**16 (20.3%)**	**18 (8.8%)**	**13 (8.1%)**	**5 (11.4%)**
**Anti-CD38–based**	0	0	14 (17.7%)	14 (6.8%)	11 (6.8%)	3 (6.8%)
DVRd	0	0	14 (17.7%)	14 (6.8%)	11 (6.8%)	3 (6.8%)
**PI + IMiD**	2 (3.2%)	0	2 (2.5%)	4 (2.0%)	2 (1.2%)	2 (4.5%)
EloRVd (trial)	2 (3.2%)	0	0	2 (1.0%)	2 (1.2%)	0
VRd + Belantamab (trial)	0	0	2 (2.5%)	2 (1.0%)	0	2 (4.5%)
**Triplet**	**47 (75.8%)**	**60 (93.8%)**	**59 (74.7%)**	**166 (81.0%)**	**132 (82.0%)**	**34 (77.3%)**
**PI + IMiD**	42 (67.7%)	49 (76.6%)	35 (44.3%)	126 (61.5%)	99 (61.5%)	27 (61.4%)
VRd	40 (64.5%)	43 (67.2%)	32 (40.5%)	115 (56.1%)	90 (55.9%)	25 (56.8%)
KRd	0	6 (9.4%)	2 (2.5%)	8 (3.9%)	8 (5.0%)	0
VTd	2 (3.2%)	0	1 (1.3%)	3 (1.5%)	1 (0.6%)	2 (4.5%)
**PI-based**	5 (8.1%)	11 (17.2%)	17 (21.5%)	33 (16.1%)	27 (16.8%)	6 (13.6%)
CyBorD	5 (8.1%)	11 (17.2%)	15 (19.0%)	31 (15.1%)	26 (16.1%)	5 (11.4%)
KCd	0	0	2 (2.5%)	2 (1.0%)	1 (0.6%)	1 (2.3%)
**Anti-CD38–based**	0	0	7 (8.9%)	7 (3.4%)	6 (3.7%)	1 (2.3%)
DRd	0	0	4 (5.1%)	4 (2.0%)	1 (0.6%)	3 (6.8%)
DVd	0	0	2 (2.5%)	2 (1.0%)	2 (1.2%)	0
DVCd	0	0	1 (1.3%)	1 (0.5%)	1 (0.6%)	0
**Doublet**	**9 (14.5%)**	**1 (1.6%)**	**2 (2.5%)**	**12 (5.9%)**	**7 (4.3%)**	**5 (11.4%)**
**PI-based**	4 (6.5%)	1 (1.6%)	1 (1.3%)	6 (2.9%)	3 (1.9%)	3 (6.8%)
Vd	4 (6.5%)	1 (1.6%)	1 (1.3%)	6 (2.9%)	3 (1.9%)	3 (6.8%)
**IMiD-based**	5 (8.1%)	0	1 (1.3%)	6 (2.9%)	4 (2.5%)	2 (4.5%)
Rd	3 (4.8%)	0	1 (1.3%)	4 (2.0%)	2 (1.2%)	2 (4.5%)
Td	2 (3.2%)	0	0	2 (1.0%)	2 (1.2%)	0
**Chemotherapy**	**2 (3.2%)**	**3 (4.7%)**	**1 (1.3%)**	**6 (2.9%)**	**6 (3.7%)**	**0**
VDPACE	0	2 (3.1%)	1 (1.3%)	3 (1.5%)	3 (1.9%)	0
D-PACE	1 (1.6%)	0	0	1 (0.5%)	1 (0.6%)	0
VTDPACE	0	1 (1.6%)	0	1 (0.5%)	1 (0.6%)	0
VDTPACE	1 (1.6%)	0	0	1 (0.5%)	1 (0.6%)	0
**Other/Novel**	**0**	**0**	**1 (1.3%)**	**1 (0.5%)**	**1 (0.6%)**	**0**
Linvoseltamab	0	0	1 (1.3%)	1 (0.5%)	1 (0.6%)	0
**Unknown/N/A**	**2 (3.2%)**	**0**	**0**	**2 (1.0%)**	**2 (1.2%)**	**0**

Abbreviations: DVRd, daratumumab/bortezomib/lenalidomide/dexamethasone; VRd, bortezomib/lenalidomide/dexamethasone; KRd, carfilzomib/lenalidomide/dexamethasone; VTd, bortezomib/thalidomide/dexamethasone; CyBorD, cyclophosphamide/bortezomib/dexamethasone; KCd, carfilzomib/cyclophosphamide/dexamethasone; DRd, daratumumab/lenalidomide/dexamethasone; DVd, daratumumab/bortezomib/dexamethasone; DVCd, daratumumab/bortezomib/cyclophosphamide/dexamethasone; Vd, bortezomib/dexamethasone; Rd, lenalidomide/dexamethasone; Td, thalidomide/dexamethasone; EloRVd, elotuzumab/VRd. Era 1 (2009–2015): PI/IMiD era; Era 2 (2016–2019): early anti-CD38 adoption; Era 3 (2020–2024): contemporary quadruplet era. Quadruplet = 3 active agents + corticosteroid; Triplet = 2 active agents + corticosteroid; Doublet = 1 active agent + corticosteroid. Two patients with unknown regimens listed separately.

**Table 3:** Second-Line Induction Therapy: Classification by Number of Agents and Specific Regimens, Stratified by Treatment Era and Age Group (N = 50).

Category/Regimen	Era 1 (2009–2015) (N = 7)	Era 2 (2016–2019) (N = 22)	Era 3 (2020–2024) (N = 21)	Overall (N = 50)	<70 Years (N = 41)	≥70 Years (N = 9)
**Quadruplet**	**0**	**0**	**2 (9.5%)**	**2 (4.0%)**	**2 (4.9%)**	**0**
**Anti-CD38 + PI + IMiD**	0	0	2 (9.5%)	2 (4.0%)	2 (4.9%)	0
DVRd	0	0	2 (9.5%)	2 (4.0%)	2 (4.9%)	0
**Triplet**	**3 (42.9%)**	**15 (68.2%)**	**16 (76.2%)**	**34 (68.0%)**	**27 (65.9%)**	**7 (77.8%)**
**PI + IMiD**	2 (28.6%)	6 (27.3%)	4 (19.0%)	12 (24.0%)	12 (29.3%)	0
VRd	2 (28.6%)	3 (13.6%)	1 (4.8%)	6 (12.0%)	6 (14.6%)	0
KRd	0	3 (13.6%)	2 (9.5%)	5 (10.0%)	5 (12.2%)	0
KPd	0	0	1 (4.8%)	1 (2.0%)	1 (2.4%)	0
**PI-based**	1 (14.3%)	4 (18.2%)	2 (9.5%)	7 (14.0%)	5 (12.2%)	2 (22.2%)
CyBorD	1 (14.3%)	3 (13.6%)	1 (4.8%)	5 (10.0%)	3 (7.3%)	2 (22.2%)
KCd	0	1 (4.5%)	1 (4.8%)	2 (4.0%)	2 (4.9%)	0
**Anti-CD38–based**	0	5 (22.7%)	10 (47.6%)	15 (30.0%)	10 (24.4%)	5 (55.6%)
DVd	0	0	5 (23.8%)	5 (10.0%)	3 (7.3%)	2 (22.2%)
DPd	0	3 (13.6%)	2 (9.5%)	5 (10.0%)	3 (7.3%)	2 (22.2%)
DKd	0	0	2 (9.5%)	2 (4.0%)	1 (2.4%)	1 (11.1%)
DRd	0	1 (4.5%)	0	1 (2.0%)	1 (2.4%)	0
DVCd → DVd	0	0	1 (4.8%)	1 (2.0%)	1 (2.4%)	0
DVd → KPd	0	1 (4.5%)	0	1 (2.0%)	1 (2.4%)	0
**Doublet**	**1 (14.3%)**	**4 (18.2%)**	**1 (4.8%)**	**6 (12.0%)**	**4 (9.8%)**	**2 (22.2%)**
**PI-based**	0	4 (18.2%)	0	4 (8.0%)	2 (4.9%)	2 (22.2%)
Vd	0	2 (9.1%)	0	2 (4.0%)	0	2 (22.2%)
Kd	0	2 (9.1%)	0	2 (4.0%)	2 (4.9%)	0
**IMiD-based**	1 (14.3%)	0	1 (4.8%)	2 (4.0%)	2 (4.9%)	0
Rd	1 (14.3%)	0	1 (4.8%)	2 (4.0%)	2 (4.9%)	0
**Chemotherapy**	**3 (42.9%)**	**2 (9.1%)**	**1 (4.8%)**	**6 (12.0%)**	**6 (14.6%)**	**0**
VRDPACE/KDPACE	3 (42.3%)	2 (9.1%)	1 (4.8%)	6 (12.0%)	6 (14.6%)	0
**Sequential/Multiple**	**0**	**1 (4.5%)**	**1 (4.8%)**	**2 (4.0%)**	**2 (4.9%)**	**0**

Abbreviations: DVRd, daratumumab/bortezomib/lenalidomide/dexamethasone; VRd, bortezomib/lenalidomide/dexamethasone; KRd, carfilzomib/lenalidomide/dexamethasone; KPd, carfilzomib/pomalidomide/dexamethasone; CyBorD, cyclophosphamide/bortezomib/dexamethasone; KCd, carfilzomib/cyclophosphamide/dexamethasone; DVd, daratumumab/bortezomib/dexamethasone; DPd, daratumumab/pomalidomide/dexamethasone; DKd, daratumumab/carfilzomib/dexamethasone; DRd, daratumumab/lenalidomide/dexamethasone; DVCd, daratumumab/bortezomib/cyclophosphamide/dexamethasone; Vd, bortezomib/dexamethasone; Kd, carfilzomib/dexamethasone; Rd, lenalidomide/dexamethasone. Sequential regimens represent patients who received multiple sequential regimens as part of second-line induction.

**Table 4:** Maintenance Therapy: Classification by Mechanism of Action and Specific Agents, Stratified by Treatment Era and Age Group (N = 147). 147/205 patients (71.7%) received maintenance therapy; 58 patients (28.3%) did not receive maintenance.

Category/Agent	Era 1 (2009–2015) (N = 50)	Era 2 (2016–2019) (N = 43)	Era 3 (2020–2024) (N = 54)	Overall (N = 147)	<70 Years (N = 123)	≥70 Years (N = 24)
**IMiD-based**	**38 (76.0%)**	**23 (53.5%)**	**17 (31.5%)**	**78 (53.1%)**	**66 (53.7%)**	**12 (50.0%)**
Lenalidomide	37 (74.0%)	21 (48.8%)	16 (29.6%)	74 (50.3%)	62 (50.4%)	12 (50.0%)
Pomalidomide	0	1 (2.3%)	0	1 (0.7%)	1 (0.8%)	0
Len → Pom	1 (2.0%)	0	0	1 (0.7%)	1 (0.8%)	0
Rd	0	0	1 (1.9%)	1 (0.7%)	1 (0.8%)	0
Pd	0	1 (2.3%)	0	1 (0.7%)	1 (0.8%)	0
**PI + IMiD combination**	**7 (14.0%)**	**12 (27.9%)**	**22 (40.7%)**	**41 (27.9%)**	**35 (28.5%)**	**6 (25.0%)**
Bortezomib + Lenalidomide	5 (10.0%)	4 (9.3%)	18 (33.3%)	27 (18.4%)	22 (17.9%)	5 (20.8%)
VRd	1 (2.0%)	3 (7.0%)	0	4 (2.7%)	4 (3.3%)	0
Carfilzomib + Lenalidomide	0	3 (7.0%)	0	3 (2.0%)	3 (2.4%)	0
KRd	0	0	2 (3.7%)	2 (1.4%)	1 (0.8%)	1 (4.2%)
Ixazomib + Rd	0	1 (2.3%)	0	1 (0.7%)	1 (0.8%)	0
Ixazomib + Pd	0	0	1 (1.9%)	1 (0.7%)	1 (0.8%)	0
KPd	0	0	1 (1.9%)	1 (0.7%)	1 (0.8%)	0
VTd → Rd	1 (2.0%)	0	0	1 (0.7%)	1 (0.8%)	0
KRd consol → K maint	0	1 (2.3%)	0	1 (0.7%)	1 (0.8%)	0
**Anti-CD38–containing**	**0**	**3 (7.0%)**	**12 (22.2%)**	**15 (10.2%)**	**11 (8.9%)**	**4 (16.7%)**
Dara + Lenalidomide	0	0	5 (9.3%)	5 (3.4%)	4 (3.3%)	1 (4.2%)
Daratumumab mono	0	1 (2.3%)	2 (3.7%)	3 (2.0%)	3 (2.4%)	0
DRd	0	0	2 (3.7%)	2 (1.4%)	1 (0.8%)	1 (4.2%)
DPd	0	2 (4.7%)	0	2 (1.4%)	2 (1.6%)	0
Dara + Pomalidomide	0	0	1 (1.9%)	1 (0.7%)	0	1 (4.2%)
Dara + Bortezomib	0	0	1 (1.9%)	1 (0.7%)	0	1 (4.2%)
DVRd consolidation	0	0	1 (1.9%)	1 (0.7%)	1 (0.8%)	0
**PI monotherapy**	**5 (10.0%)**	**5 (11.6%)**	**1 (1.9%)**	**11 (7.5%)**	**11 (8.9%)**	**0**
Bortezomib	3 (6.0%)	2 (4.7%)	1 (1.9%)	6 (4.1%)	6 (4.9%)	0
Ixazomib	1 (2.0%)	2 (4.7%)	0	3 (2.0%)	3 (2.4%)	0
Kd	1 (2.0%)	1 (2.3%)	0	2 (1.4%)	2 (1.6%)	0
** Other/Novel**	**0**	**0**	**2 (3.7%)**	**2 (1.4%)**	**0**	**2 (8.3%)**
Belantamab + Len (trial)	0	0	2 (3.7%)	2 (1.4%)	0	2 (8.3%)

Abbreviations: IMiD, immunomodulatory drug; PI, proteasome inhibitor; Len, lenalidomide; Pom, pomalidomide; Dara, daratumumab; VRd, bortezomib/lenalidomide/dexamethasone; KRd, carfilzomib/lenalidomide/dexamethasone; DRd, daratumumab/lenalidomide/dexamethasone; DPd, daratumumab/pomalidomide/dexamethasone; KPd, carfilzomib/pomalidomide/dexamethasone; DVRd, daratumumab/bortezomib/lenalidomide/dexamethasone; Kd, carfilzomib/dexamethasone. PI + IMiD category includes doublets (e.g., bortezomib + lenalidomide) and triplets (e.g., VRd). Anti-CD38–containing regimens were absent in Era 1 and emerged primarily in Era 3. PI monotherapy was used exclusively in patients <70 years (11/123 vs. 0/24).

**Table 5:** Transplant Characteristics of Patients with High-Risk Multiple Myeloma by Age Group.

Characteristic	Overall (N = 205)	<70 Years (N = 161)	≥70 Years (N = 44)
Patients undergoing ASCT	154 (75.1%)	135 (83.9%)	19 (43.2%)
Autologous SCT	153 (99.4%)	134 (99.3%)	19 (100.0%)
Allogeneic SCT	1 (0.6%)	1 (0.7%)	0 (0.0%)
Melphalan conditioning dose			
200 mg/m^2^	124 (80.5%)	115 (85.2%)	9 (47.4%)
140 mg/m^2^	25 (16.2%)	15 (11.1%)	10 (52.6%)
100 mg/m^2^	1 (0.6%)	1 (0.7%)	0 (0.0%)
BuMel regimen	1 (0.6%)	1 (0.7%)	0 (0.0%)
N/A (includes allogeneic)	3 (1.9%)	3 (2.2%)	0 (0.0%)
Tandem ASCT			
Yes	14 (9.1%)	14 (10.4%)	0 (0.0%)
No	137 (89.0%)	118 (87.4%)	19 (100.0%)
N/A	3 (1.9%)	3 (2.2%)	0 (0.0%)

“Patients undergoing ASCT” row uses total cohort as denominator (N = 205; <70 N = 161; ≥70 N = 44). Auto/Allo, Melphalan dose, and Tandem rows use ASCT patients as denominator (N = 154; <70 N = 135; ≥70 N = 19). One patient received allogeneic SCT; melphalan dose recorded as “Allo/SCT” and classified under N/A. Abbreviations: ASCT, autologous stem cell transplant; SCT, stem cell transplant; BuMel, busulfan/melphalan; N/A, not applicable (i.e., variable not applicable to patients who did not undergo ASCT).

**Table 6:** Response Outcomes by Treatment Phase in Patients with High-Risk Multiple Myeloma.

Response Category	1st Induction (N = 205)	2nd Induction (N = 50)	Post-ASCT (N = 154)	Maintenance 6 mo (N = 147)	Maintenance 12 mo (N = 147)
ORR (≥PR)	172 (83.9%)	45 (90.0%)	136 (88.3%)	97 (66.0%)	74 (50.3%)
≥VGPR	115 (56.1%)	36 (72.0%)	129 (83.8%)	96 (65.3%)	73 (49.7%)
CR/sCR	45 (22.0%)	13 (26.0%)	78 (50.6%)	68 (46.3%)	57 (38.8%)
sCR	10 (4.9%)	2 (4.0%)	26 (16.9%)	24 (16.3%)	18 (12.2%)
CR	35 (17.1%)	11 (22.0%)	52 (33.8%)	44 (29.9%)	39 (26.5%)
VGPR	70 (34.1%)	23 (46.0%)	51 (33.1%)	28 (19.0%)	16 (10.9%)
PR	57 (27.8%)	9 (18.0%)	7 (4.5%)	1 (0.7%)	1 (0.7%)
SD	3 (1.5%)	1 (2.0%)	0 (0.0%)	2 (1.4%)	2 (1.4%)
PD	17 (8.3%)	3 (6.0%)	11 (7.1%)	22 (15.0%)	28 (19.0%)
Not evaluable	13 (6.3%)	1 (2.0%)	7 (4.5%)	26 (17.7%)	43 (29.3%)

## Data Availability

The data presented in this study are available upon reasonable request from the corresponding author. The data are not publicly available because of privacy restrictions.
